# Tumor antigen-loaded AAV vaccine drives protective immunity in a melanoma animal model

**DOI:** 10.1016/j.omtm.2023.01.006

**Published:** 2023-02-02

**Authors:** Karina Krotova, Hisae Kuoch (Yoshitomi), Colin Caine, George Aslanidi

**Affiliations:** 1 Hormel Institute, University of Minnesota, 801 16th Avenue NE, Austin, MN 55912, USA; 2 Masonic Cancer Center, Minneapolis, MN 55912, USA

**Keywords:** adeno-associated virus, cancer vaccine, self-antigen, antigen-specific T cell, protective immune response, syngeneic animal model

## Abstract

We previously described therapeutic opportunities provided by capsid- and expression cassette-optimized adeno-associated virus serotype 6 (AAV6) vectors to suppress tumor growth in both solid and metastatic mouse models by using artificial ovalbumin (OVA) immunogen. In the current study, we further elucidated the mechanism of function of a novel AAV-based vaccine loaded with the melanoma tumor-associated antigens premelanosome protein gp100, tyrosinase (Tyr), tyrosinase-related protein 1 (TRP1), and dopachrome tautomerase (TRP2). We showed that the AAV6-based vaccine creates cellular and humoral antigen-specific responses, while antigen expression at the site of vaccine injection was temporal, and the clearance of antigen coincided with T cell infiltration. Our data revealed the superior protective immune response of optimized AAV6-TRP1 compared with other self-antigens in a disease-free mouse model. We further assessed the ability of AAV6-TRP1 to protect animals from metastatic spread in the lungs and to extend animal survival by inhibiting solid tumor growth. Flow cytometry-based analysis indicated significant infiltration of CD8^+^ T cells and natural killer (NK) cells in the tumor site, as well as changes in the polarization of intratumoral macrophages. Altogether, our data strongly support the use of optimized AAV vectors for cancer vaccine development.

## Graphical abstract

**Figure:**
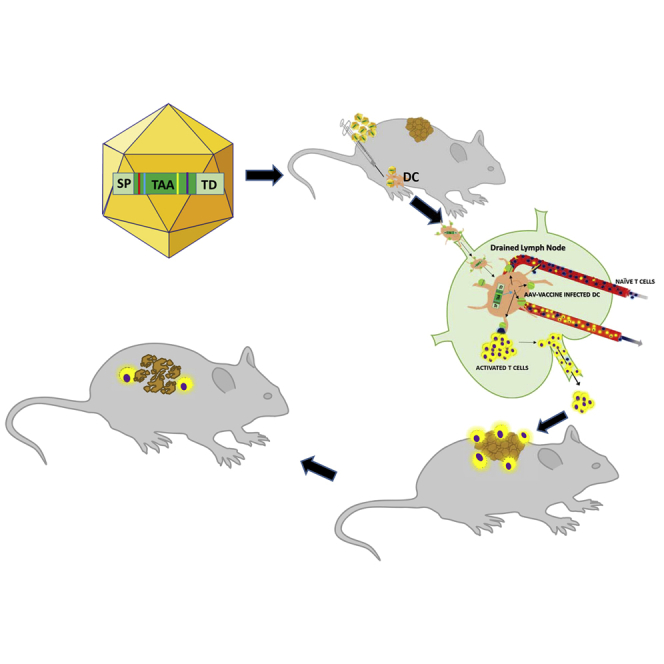

## Teaser

The major challenge with the cancer vaccine is the lack of an appropriate “danger signal” for proper “education” of the immune system to exclusively attack cancer cells. We developed an effective vaccine delivery platform and tested our vaccine on the animal model that mimics human melanoma.

## Introduction

Adeno-associated virus (AAV) vectors are widely used in preclinical and clinical gene therapy for the stable expression of a therapeutic transgene because of its safety profile.^1^ The use of AAV as a platform for vaccines has attracted less attention since the first studies in mice suggested that AAV vectors are poor transducers of antigen-presenting cells (APCs) and, consequently, generate functionally impaired antigen-specific CD8^+^ T cells.^2^ The discovery of new serotypes and progress in vector bioengineering renewed interest in developing AAV vectors suitable for vaccine applications.^3^^,^^4^^,^^5^ In addition, the COVID-19 era has provided an incredible boost^6^^,^^7^^,^^8^ in the development of different viral platforms for vaccine application, including AAV.^9^ Multiple studies have shown that the immune response to transgenes delivered by AAV depends on multiple factors, including the intrinsic immunogenicity of the transgene, preexisting immunity against the transgene, ability to activate CD4^+^ T helper cells, route of delivery, and AAV serotype and dose.^10^^,^^11^^,^^12^^,^^13^^,^^14^ More importantly, the immunogenicity of transgenes delivered by AAV vectors can be manipulated in both directions: to reduce the immune response for gene therapy applications or to increase it for vaccine formulations.

Vaccine development requires vectors that target APCs and are able to induce a strong immune response, while transgene expression should be temporal. To meet these requirements, several different approaches were previously explored, including capsid bioengineering and antigen (Ag) modifications. For example, the hybrid from two related rhesus macaque isolates, AAVrh32.33, aligned with these requirements and is considered a suitable AAV strain for vaccine development.^12^ Compared with other unmodified AAV serotypes, AAVrh32.33 generates functional Ag-specific CD8^+^ T cells that successfully clear transgenes from injected muscles. Importantly, the generation of Ag-specific CD8^+^ T cells was dependent on CD4^+^ T helper cells stimulated directly by AAV capsid epitopes and on the activation of CD40 L^+^ and CD28^+^ costimulatory molecules.^15^^,^^16^ Other groups have shown that the AAV1 serotype can be used for vaccine development.^13^^,^^17^^,^^18^ While AAV1 is generally a poor transducer of APCs, the intradermal route of immunization activates cross-presenting APCs against transgenes expressed in mouse skin. The combination of intradermal injection and fusion of Ag with male HY Ag epitopes, which additionally provides CD4^+^ T helper cells in female mice, ensured the generation of functional Ag-specific cytotoxic T cells against model Ag ovalbumin (OVA).

We previously showed that AAV6 can directly target APCs both *in vitro* and *in vivo* and can be used for vaccine applications.^19^^,^^20^ Furthermore, the rational design of the capsid by introducing mutation S663V improved intracellular trafficking of AAV6 vectors, which provides an early onset of gene expression and increases the overall quantity of Ag production.^20^ Based on self-complementary AAV6-S663V, we designed a vaccine by flanking highly immunogenic Ag OVA with trafficking signals of the major histocompatibility complex (MHC) class I molecule to facilitate the generation of CD4^+^ T helper cells and showed that this vaccine induced strong cellular and humoral immune responses.^19^

In the present study, we used the same design to develop an optimized AAV6-based vaccine against low immunogenic melanoma tumor-associated self-Ags. We show that the vaccine generates Ag-specific CD8^+^ T cells and that the transgene is quickly cleared from the injected muscle at the time the muscle is infiltrated with T cells. We also showed that a single intramuscular injection of the AAV-based vaccine provides protection in a prophylactic tumor model and delays tumor growth in a therapeutic model of mouse melanoma. Our data suggest that the repertoire of diseases possibly treated with AAV-based vaccines can be extended to cancers in cases when appropriate Ag(s) are available as a target.

## Results

### Analysis of AAV-based vaccines against four tumor-associated Ags (TAAs) in a prophylactic tumor-challenged model

**Figure 1:**
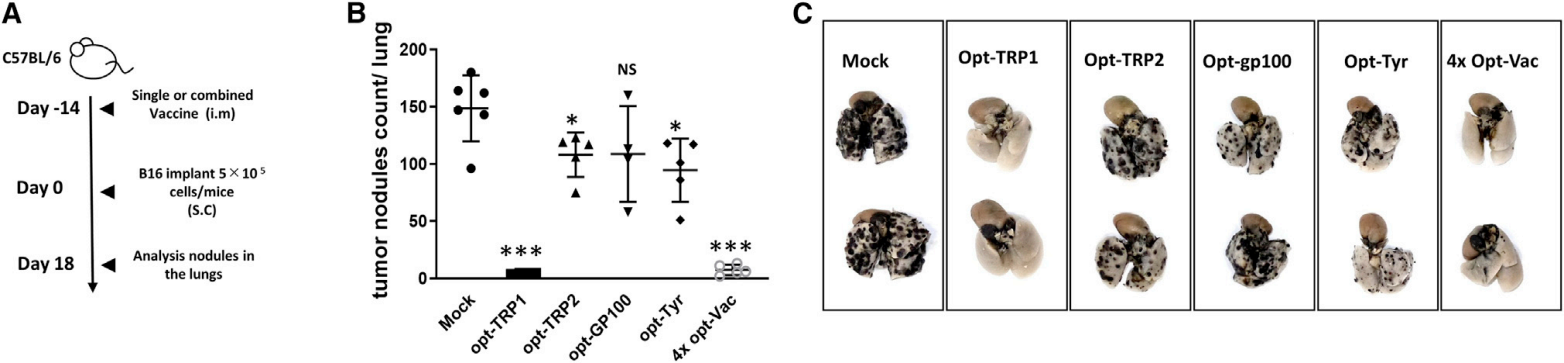
AAV vaccine induces a strong antitumoral response in a prophylactic model (A) Scheme of the treatment schedule. C57BL6 mice were vaccinated through a single intramuscular injection with AAV-based vaccines against individual transgenes or with 4 vaccines combined in one shot to create a multivalent vaccine. Two weeks later, vaccinated and control mice were transplanted with B16F10 melanoma cells intravenously. Eighteen days later, the mice were sacrificed, and melanoma nodules in the lungs were counted. (B) Summary of melanoma nodule count and (C) representative images of lungs at the time of count. ∗p < 0.05, ∗∗∗p < 0.001, mock vs. vaccine treatment.

The initial evaluation of the immune response generated by AAV-based vaccines was performed on a prophylactic tumor-challenged mouse model. Mice were first vaccinated by a single intramuscular injection, and 2 weeks later, at the optimal time of Ag-specific cytotoxic T lymphocyte (CTL) development,^19^ mice were transplanted with B16 cells through intravenous injection (Figure 1A). This model allows estimation of the strength of vaccine-induced CTLs to kill tumor cells without interference from the tumor microenvironment (TME). The vaccine was formulated as AAV loaded with a single TAA against one of four well-described TAAs: opt-TRP-1, opt-TRP-2, opt-gp-100, and opt-Tyr at a dose of 1 × 10^10^ vg/mouse. We then combined individual vaccines at the same titer (1 × 10^10^ vg/mouse) in one shot and defined it as a multivalent vaccine 4× opt-Vac (4 × 10^10^ total vg/mouse). AAV vaccines based on each of the four TAAs provided different levels of protection (Figures 1B and 1C). Opt-TRP1 showed the strongest protection, with 0–10 tumor nodules per lung compared with more than 150 in mock-treated mice, and opt-Gp100 was the weakest, with no significant difference in the number of tumor nodules compared with mock-treated mice. The protection from the combinatorial vaccine 4× opt-Vac was robust and comparable to the therapeutic effect of opt-TRP1 alone (˂10 tumor nodules per lung).

**Figure 2:**
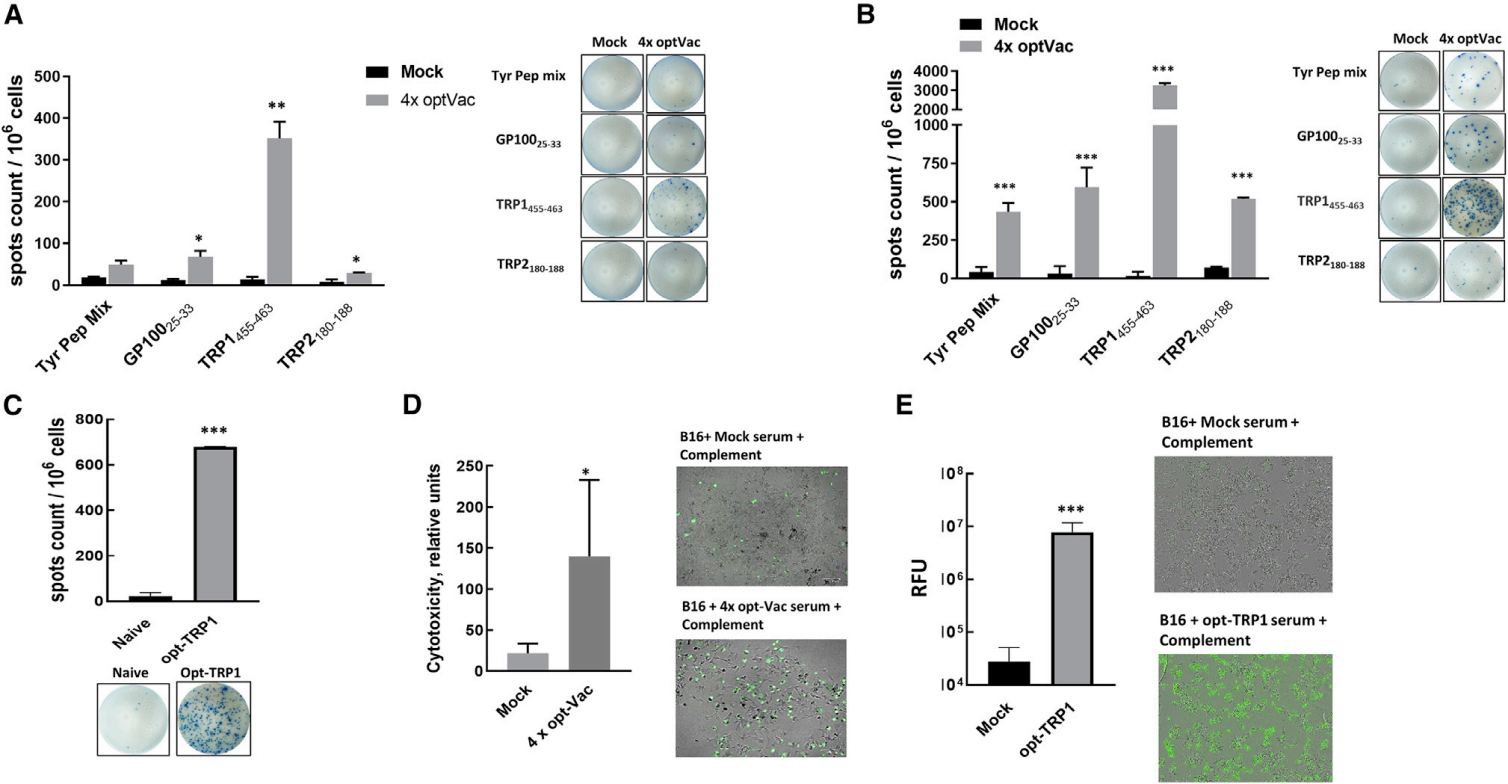
Antigen-specific response created by AAV-based vaccine Mice were vaccinated with an AAV vaccine composed of four AAV vaccines against individual melanoma-associated antigens combined in one-shot optVac (A, B, and D) or with opt-TRP1 (C and E). Four weeks after vaccination, mice were sacrificed, and plasma and splenocytes were collected for analysis. (A and C) Fresh splenocytes were restimulated with the corresponding peptides and analyzed by IFNγ ELISPOT. At least three mice per group were analyzed. (B) Splenocytes were stimulated with the indicated peptides, cultured for 7 days before restimulation, and subjected to ELISPOT analysis. (D and E) Complement-dependent cytotoxicity (CDC) assay. The complement is able to kill tumor cells in the presence of plasma from mice vaccinated against TAA but not mock-vaccinated mice. Cell death was measured by the fluorescence of Cytotox green bound to nucleic acids of dying cells. For each group, serum from four mice was analyzed. ∗p˂ 0.05, ∗∗p˂ 0.01, ∗∗∗p˂ 0.001, mock vs. vaccine treatment.

The generation of CTLs against each TAA was analyzed by the ability of splenocytes from opt-Vac-vaccinated mice to produce interferon γ (IFNγ) measured by enzyme-linked immunospot assay (ELISPOT). Analysis was performed on fresh splenocytes to capture high-magnitude T cells and on splenocytes cultured for 7 days with stimulation of specific peptide(s) to enhance the response. We found that fresh splenocytes from opt-Vac-immunized mice were able to produce high levels of IFNγ in response to stimulation with TRP-1 peptide (more than 100 spots), while peptides from other Ags were able to stimulate IFNγ production from fewer cells (Figure 2A). The highest number of IFNγ-producing cells induced by the TRP-1 peptide was in agreement with the results of the best protection provided by opt-TRP1 against B16F10 lung tumor nodules. In contrast to fresh splenocytes, all four Ags produced high levels of IFNγ after culturing for an additional 7 days (Figure 2B). These data indicate that vaccination was able to break self-tolerance against all four Ags with the highest number of generated CTLs against TRP-1. We further confirmed that vaccination with opt-TRP1 alone also generated high levels of IFNγ-producing splenocytes (Figure 2C).

We previously showed that in addition to the cellular response, the AAV vaccine is able to generate a humoral response when OVA is used as an Ag.^19^ To test whether the vaccine can induce antibodies against self-Ags, the plasma from immunized animals was tested in a complement-dependent cytotoxicity assay. We found that the plasma from mice immunized with multivalent opt-vaccine or single opt-TRP1 was superior in mediating complement-induced killing of B16F10 cells compared with plasma from mice immunized with nonrelevant AAV (Figures 2D and 2E).

While the multivalent vaccine against all four TAAs did not improve protection over the strongest TAA alone (opt-TRP1), it is important to emphasize that our results showed that different Ags delivered in the single shot do not compete with each other and do not reduce the immunogenicity of the strongest one. This is an important observation for the application of off-shelf vaccines that are intended to be used in heterogeneous patient populations where immunodominant Ags vary depending on HLA haplotype. In that case, the combination of different TAAs would provide the best opportunity to target the most immunogenic Ag.

Since prophylactic vaccination with opt-TRP1 provided the best protection against B16F10 tumor development compared with other Ag formulations in the current study, the next experiments were performed with opt-TRP1 alone unless indicated otherwise.

### Immunization with AAV vaccine induces short-term transgene expression at the site of injection, which is cleared in a timely manner and coincides with T cell infiltration

Immunization against cancer is aimed at generating cytotoxic T cells against tumor Ag delivered by the vaccine. However, if the presence of Ag delivered by the vaccine persists for a long period of time at the site of injection, it can traffic Ag-specific cytotoxic CD8^+^ T cells to the site of injection instead of to the tumor, resulting in chronic inflammation and exhaustion of T cells.^21^ Therefore, one of the important prerequisites for successful vaccine formulation is the prompt clearance of the transgene from the site of injection.

**Figure 3:**
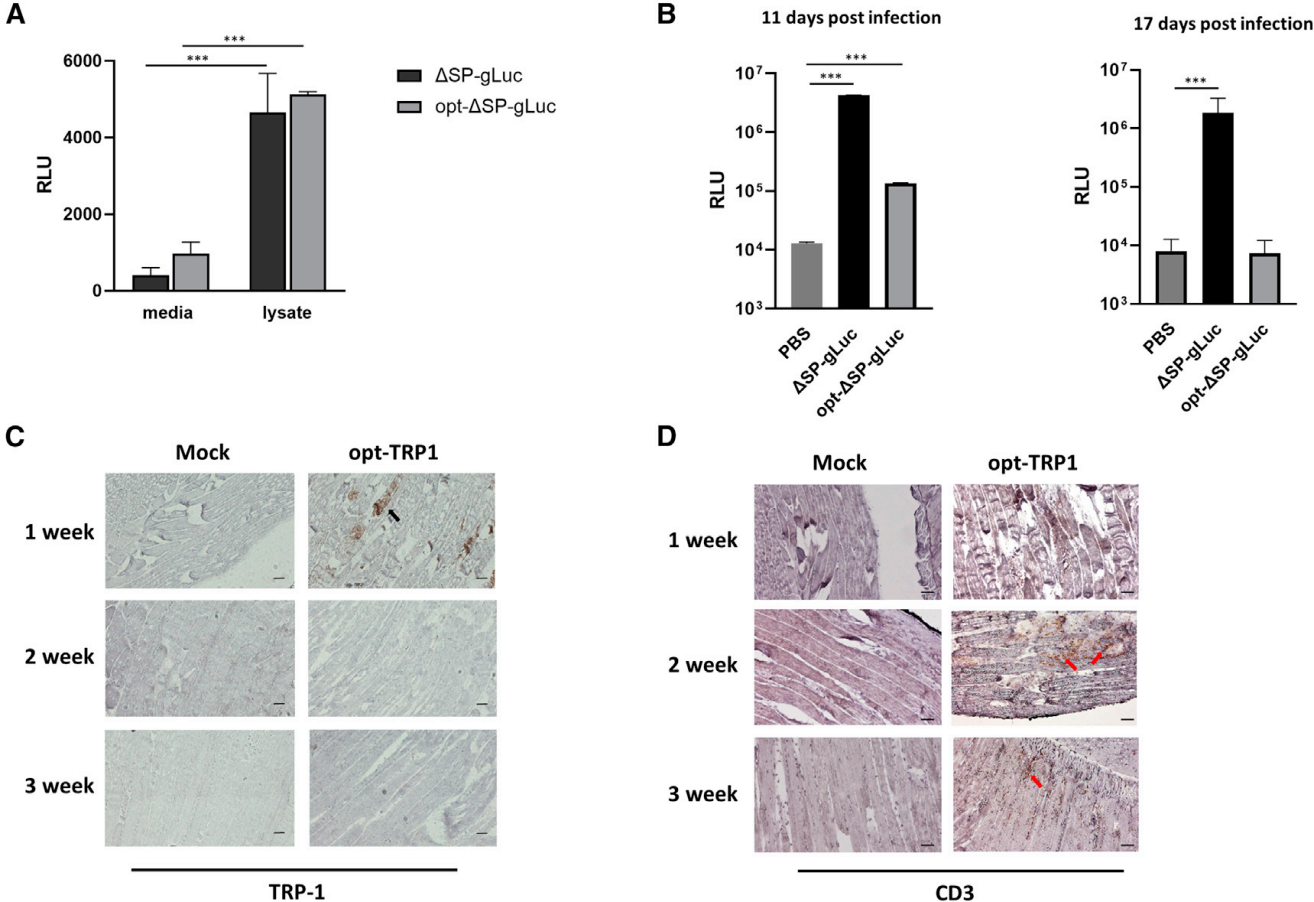
Retention of antigen expression at the site of vaccination (A) Luciferase activity in HEK293 cells 48 h post infection. The activity in cell lysates was much higher than the activity in conditioned media, indicating that the signal peptide Gaussia luciferase was retained in the cells. The luciferase activity between ΔSP-gLuc and opt-ΔSP-gLuc constructs was comparable in both conditioned media and cell lysates. (B) Luciferase activity retained in muscles injected with ΔSP-gLuc but not in opt-ΔSP-gLuc. Significant luciferase activity was observed for both constructs in muscles collected 11 days post-injection. However, at 17 days post-injection, the luciferase activity remained only in muscles injected with ΔSP-gLuc and completely disappeared in muscle injected with opt-ΔSP-gLuc. ∗∗∗p˂ 0.001. (C and D) IHC of muscles injected with opt-TRP1. (C) Expression of TRP-1 as a transgene in muscle injected with opt-TRP1 analyzed 1–3 weeks post-injection. Positive staining with anti-TRP1 antibodies was observed at week 1 after injection and disappeared at weeks 2 and 3. (D) The disappearance of TRP1 expression in injected muscles coincides with the infiltration of T cells. Staining with anti-CD3 antibodies indicated that there were no noticeable T cells at week 1 after injection, but muscles infiltrated with T cells at weeks 2 and 3.

To estimate the effect of our vaccine design on the persistence of transgene expression, we used Gaussia luciferase (gLuc) with a deleted signal peptide (ΔSP-gLuc) as the reporter gene. ΔSP-gLuc was cloned in AAV6-S663V without modifications or in a vaccine form flanked with MHC class I leader peptide and trafficking domain (opt-ΔSP-gLuc). The activity of the constructs was first confirmed in HEK293 cells, and both constructs demonstrated equal luciferase activity in cellular lysates (Figure 3A). Then, luciferase activity was studied *in vivo* after ΔSP-gLuc and opt-ΔSP-gLuc injection in mouse gastrocnemius muscle at different time points. While muscles injected with ΔSP-gLuc demonstrated stable luciferase activity over the period of the experiment, the luciferase activity from opt-ΔSP-gLuc was gradually reduced and completely disappeared at 17 days post-injection (Figure 3B). Therefore, the modification of transgene with MHC class I leader peptide and trafficking domain is enough to change the transgene expression from stable to temporal. We and others showed previously that this modification is responsible for increased generation of Ag-specific CD4^+^ T helper cells, which, in turn, increase the cytotoxic capacity of Ag-specific CD8^+^ T cells.^19^^,^^22^

In a separate experiment, we analyzed the expression of the transgene in muscles injected with the opt-TRP1 vaccine. Immunohistochemistry with anti-TRP-1 antibodies demonstrated TRP-1 expression in injected muscles 1 week after immunization and no evidence of TRP-1 expression 2 and 3 weeks later (Figure 3C). At the same time, the infiltration of T cells in injected muscles became prominent at 2 weeks after the injection, and T cells could still be observed at 3 weeks (Figure 3D). These data demonstrate that the transgene delivered by our designed AAV vaccine was cleared from injected muscle after short-term expression.

### Analysis of AAV-based vaccines against four different TAAs in the treatment model

**Figure 4:**
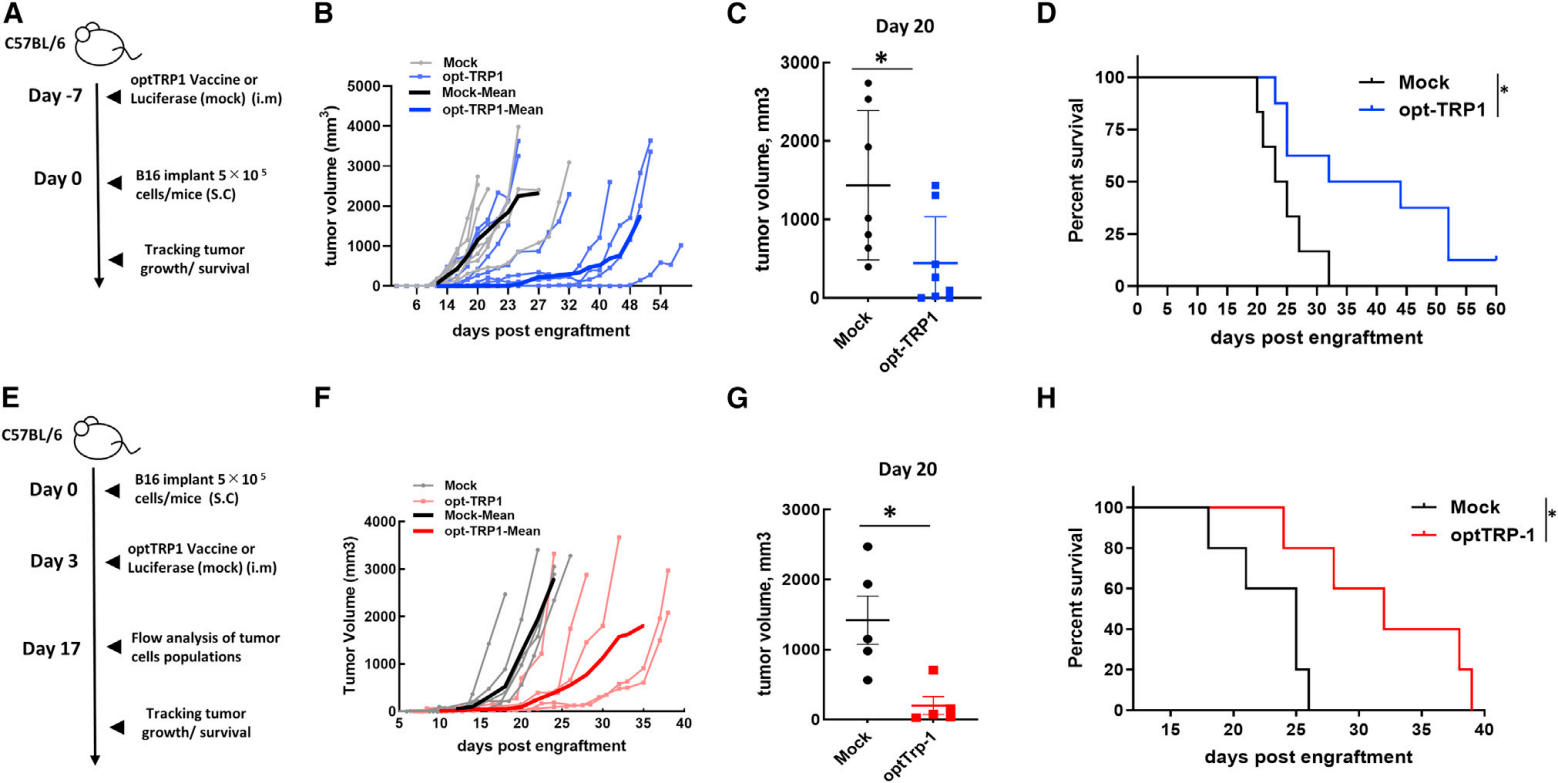
Vaccination significantly decreases melanoma tumor growth in treatment models Two schemes of treatment were used. Mice were vaccinated with a single intramuscular shot of opt-TRP1 7 days before tumor transplantation (A) or 3 days after tumor transplantation (E). (B and F) Individual and mean for each group tumor growth curves. (C and G) Tumor volume in treatment groups at day 20 after tumor implantation. p < 0.05 mock-treated vs. vaccinated. (D and H) Cumulative Kaplan-Meier survival curves demonstrate prolonged survival of mice treated with vaccine. ∗p < 0.05 mock-treated vs. vaccinated.

The B16F10 melanoma is a fast-growing aggressive tumor with a short window of time to apply treatments, while the AAV vaccine requires at least 2 weeks to generate a sufficient CTL response. To study the efficiency of the AAV-based vaccine in a melanoma treatment model and have adequate time to develop the CTL response, we utilized two different protocols with modifications at the time point of vaccine administration. In the first protocol, mice were vaccinated 7 days before subcutaneous tumor engraftment, and in the second protocol, mice were vaccinated 3 days after tumor implantation. In both protocols, tumor cells were injected prior to the appearance of CTLs generated by the vaccine, which, in general, takes 2 weeks. Our data suggest that vaccination with opt-TRP1 significantly delayed tumor growth and increased survival time in both protocols (Figure 4). However, in the first protocol, the difference in survival was, on average, 20 days between the control and vaccinated groups and 13 days in the second protocol. This finding confirmed the importance of appropriate timing in vaccine administration since, in the second protocol, CTLs appeared when tumors were in a significantly more advanced stage and, thus, harder to treat.

**Figure 5:**
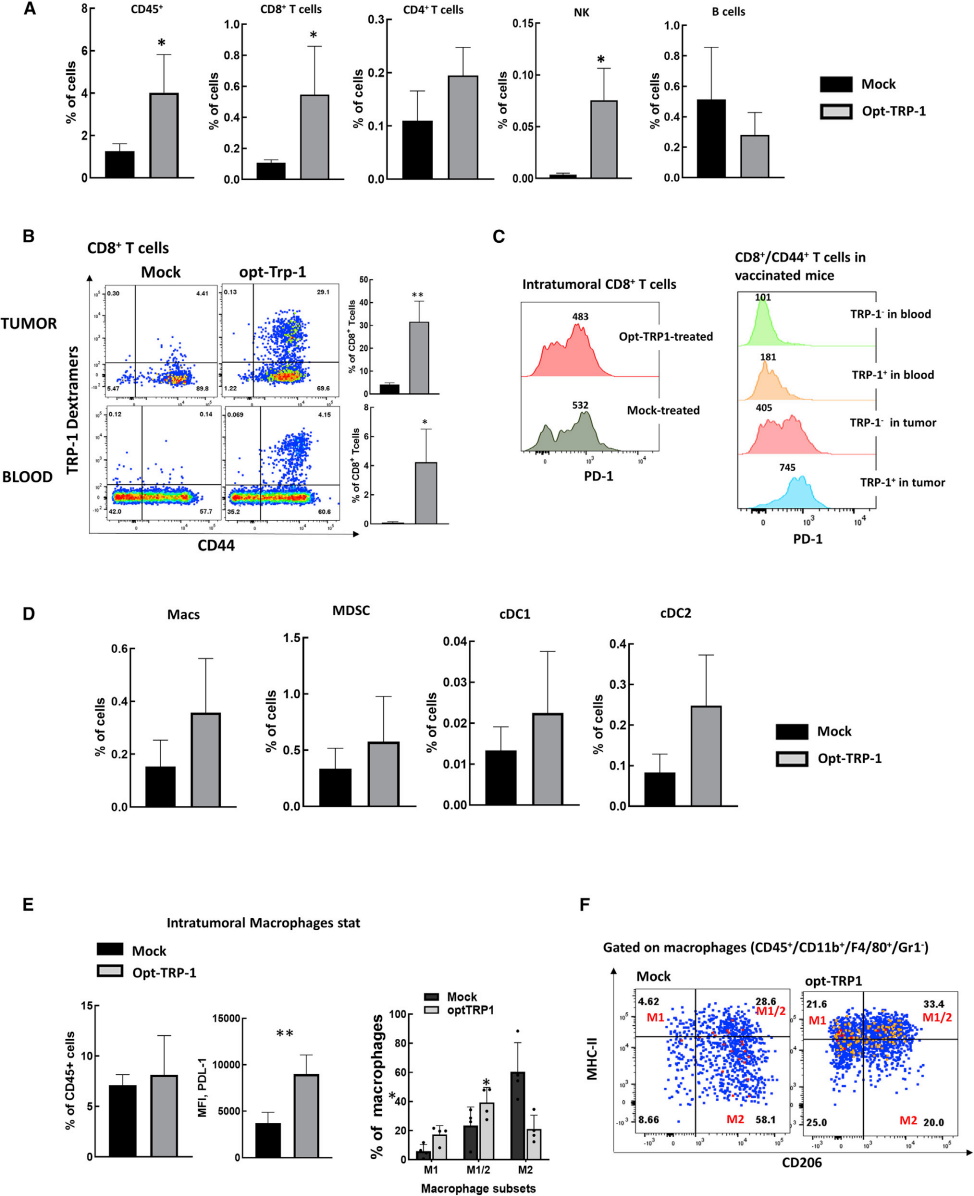
Tumor infiltration by immune cells in response to opt-TRP1 vaccination (A) Flow analysis of intratumoral lymphocytes in mice vaccinated with opt-TRP1 compared with mock-treated tumor-bearing mice. ∗p < 0.05 mock-treated vs. vaccinated. (B) Flow analysis of anti-TRP1 CD8^+^ T cell accumulation in tumors (top panel) and blood (bottom panel) of vaccinated mice. Representative plots and statistical analysis. ∗p < 0.05, ∗∗p < 0.01 mock-treated vs. vaccinated. (C) Flow analysis of PD-1 expression on CD8^+^ T cells in tumor and blood of vaccinated and mock-treated mice. (D) Changes in the numbers of intratumoral immune cells of myeloid lineage after vaccination: macrophages (Macs), MDSCs, and dendritic cells (DCs). (E) Macrophages became more M1 polarized and expressed higher levels of PD-L1 ∗p < 0.05, ∗∗p < 0.01 mock-treated vs. vaccinated. (F) Representative plots of macrophage polarization.

Next, we analyzed the composition of immune cells in the TME at 2 weeks after vaccination (Figure 5). We found that vaccination increased the total number of CD45^+^ immune cells, and a statistically significant increase was observed in populations of CD8^+^ T and NK cells but not of CD4^+^ T or B cells. Dextramer staining showed that TRP1-specific CD8^+^ T cells accumulated in tumors and presented a significant portion, up to 30%, of intratumoral CTLs. Additionally, the presence of TRP1-specific CD8^+^ T cells in the tumor was much higher than that in the blood circulation of the same mouse (Figure 5B). Analysis of PD-1 expression showed that TRP1-specific CTLs expressed higher levels of PD-1 in the tumor than in blood. This could indicate the recognition of tumor Ags by TRP1-specific CTLs, which results in the process of activation/exhaustion upon entering the tumor (Figure 5C). At the same time, only a portion of TRP1^−^ CTLs are PD1^+^ both in vaccinated and mock-treated animals. We also analyzed the changes in intratumoral populations of myeloid origin. Macrophages (Macs) were defined as CD45^+^/CD11b^+^/Gr-1^−^/F4/80^+^, myeloid-derived suppressor cells (MDSCs) as CD45^+^/MHC class II^−^/CD11b^+^/Gr-1^+^, conventional dendritic cells 1 (DC1s) as CD45^+^/CD11c^+^/MHC class II ^high^/CD11b^−^/CD103^+^, and cDC2s as CD45^+^/CD11c^+^/MHC class II^high^/CD11b^+^/CD103^+^. The difference in the levels of populations of myeloid origin varied greatly between tumors and did not reach statistical significance between the vaccine-treated and mock-treated groups (Figure 5D). However, we observed statistically significant changes in macrophage phenotypes. The intratumoral macrophages expressed significantly higher levels of PDL-1 in vaccinated animals. We also analyzed the expression of CD206, which is generally considered a marker of M2 macrophages, and the expression of MHC class II, which is upregulated on M1 macrophages.^23^ The majority of macrophages in mock-treated tumors were CD206^+^/MHC class II^−^, and after vaccination, macrophages expressed higher levels of MHC class II and reduced levels of CD206 (Figures 5E and 5F). These data show that vaccination induces global changes in tumor-infiltrating immune cells; however, defining the role of these changes requires additional experiments.

## Discussion

AAV vectors have a proven record of success for single-gene diseases, although they can also be used for cancer research and possible treatment.^24^^,^^25^ Specifically, the AAV6 vector and its modifications, unlike some other AAV serotypes, are suitable for targeting lymphoid^26^^,^^27^^,^^28^ and myeloid cells^19^^,^^20^^,^^29^^,^^30^^,^^31^ for immunotherapy.

In this study, we assessed the ability of AAV6-based vaccines to target specific TAAs using mouse melanoma B16F10 cells as a model system. We previously showed that the formulation of vaccine we designed can directly target APCs and, in addition to Ag-specific CD8^+^ T cells, have the ability to generate high levels of Ag-specific CD4^+^ T cells.^19^ TAAs are nonmutated self-Ags overexpressed by tumor cells and are attractive targets for off-shelf antitumor vaccines because they are broadly expressed compared with neoantigens, which are unique to individual tumors. At the same time, TAA immunogenicity is low, as high-affinity T cells against self-proteins are deleted from the immune repertoire during development.^32^^,^^33^ Therefore, for the successful targeting of TAA by vaccination, the immunotolerance barrier should be broken.

We applied several approaches to increase the immunogenicity of self-Ags, including the use of congenic sequences and the introduction of point mutations. In addition, we use partial sequences of Ags. It was proposed that the truncated version of Ag(s) is directed to degradation in high proportion during translation and might be better processed by APCs, which leads to improved activation of specific immune responses^34^ (the modifications applied for each vaccine design are summarized in Table 1). As a result, the vaccines against the four melanoma Ags we designed were able to generate cytotoxic T cells, although at varying strengths. Nearly complete protection from growth of the lung melanoma nodules was achieved with the vaccine encoding TRP-1, while the vaccine with gp100 as a target provided the lowest level of protection. Importantly, the vaccine not only created CTLs, which were estimated by the ability to produce IFNγ after restimulation, but also generated Ag-specific antibodies that were able to facilitate complement-dependent killing of B16F10 cells, as shown by an *in vitro* assay. The activation of both cellular and humoral immunity against TAAs by vaccination significantly increases the chances of successful tumor eradication. The majority of the AAV-based vaccines reported thus far have shown strong cellular and humoral responses against viral^12^ or model Ags,^11^^,^^13^^,^^16^ which are considerably strong immunogens. Hence, it is an important observation that our AAV vaccines successfully created both humoral and cellular immune responses against melanoma TAAs with relatively low immunogenicity.

**Table 1:** Nomenclature of AAV constructs

AAV vectors construct abbreviation used in article	Full name of AAV vector constructs	Brief description
AAV6	wild-type AAV6	unmodified capsid
663-AAV6	AAV6-S663V	capsid-optimized AAV6 with substitution of serine at position 663 to valine
opt-Tyr	AAV6-S663V/self-complementary-CMV-Sec-hTyrosinase-MITD	capsid-optimized AAV6 expressing truncated human tyrosinase (2–276 aa) flanked by human MHC class I N-terminal leader peptide (Sec, 78 bp) and C-terminal trafficking signal (MITD, 168 bp)
opt-gp100	AAV6-S663V/self-complementary-CMV-Sec-hgp100-MITD	capsid-optimized AAV6 expressing truncated human gp100 (2–271 aa) flanked by human MHC class I N-terminal leader peptide (Sec, 78 bp) and C-terminal trafficking signal (MITD, 168 bp)
opt-TRP2	AAV6-S663V/self-complementary-CMV-Sec-mTRP2-MITD	capsid-optimized AAV6 expressing truncated mouse TRP2 (2–274 aa) flanked by human MHC class I N-terminal leader peptide (Sec, 78 bp) and C-terminal trafficking signal (MITD, 168 bp)
opt-TRP1	AAV6-S663V/self-complementary-CMV-Sec-mTRP1 -MITD	capsid-optimized AAV6 expressing truncated mouse TRP1 (257–506 aa), with substitution of alanine at position 463 to methionine^35^ and flanked by human MHC class I N-terminal leader peptide (Sec, 78 bp) and C-terminal trafficking signal (MITD, 168 bp)
ΔSP-gLuc	AAV6-S663V/self-complementary-CMV-ΔSP- Gaussia luciferase	capsid-optimized AAV6 expressing truncated Gaussia luciferase with deleted N-terminal signal peptide (2–16 aa)
opt-ΔSP-gLuc	AAV6-S663V/self-complementary-CMV- Sec-ΔSP-Gaussia-MITD	capsid-optimized AAV6 expressing truncated Gaussia luciferase with deleted N-terminal signal peptide (2–16 aa) flanked by human MHC class I N-terminal leader peptide (Sec, 78 bp) and C-terminal trafficking signal (MITD, 168 bp)
Mock	AAV6-S663V-fluc	capsid-optimized AAV6 expressing firefly luciferase

An early report that analyzed transgene-specific CD8^+^ T cells primed with an AAV vaccine found that vaccines based on AAV serotypes 1, 2, 5, 7, 8, and 9 induced partially dysfunctional CTLs against transgenes.^2^ These CTLs were not able to expand or produce IFNγ in response to restimulation and did not protect against the model pathogen. This functional impairment was caused by persistence of transgene expression, and while CTLs infiltrated infected muscle, they were unable to clear the transgene and sequentially underwent programmed death.^2^^,^^21^ Additionally, the poor transduction of DCs by some AAVs might be partially due to inefficient vector uncoating, which limits their transduction capabilities.^36^

In contrast, our vaccines, which were established based on an optimized AAV6 vector, provided CTL-based protection from tumor development in prevaccinated mice, both with model Ag OVA^19^ and with TAA. Moreover, we observed significant expansion in the number of IFNγ-producing CTLs after the restimulation of splenocytes with Ag for an additional 7 days before analysis (Figure 2). It is important to note that the reports of successful Ag-specific CTLs generation by AVV-based vaccines emphasized the importance of the presence of CD4^+^ T helper cells.^37^^,^^38^^,^^39^ These CD4^+^ T helper cells could be induced by the capsid itself^15^ through the addition of an irrelevant MHC class II immunodominant epitope to the transgene^11^ or, in our case, by directing the posttranslational processing of the transgene to be presented on MHC class II.^19^

Importantly, we also showed that these modifications in the capsid and expression cassette of our vaccine changed the fate of the transgene at the site of injection by using luciferase. Instead of stable expression, as is the case with unmodified luciferase, the expression of luciferase from the optimized construct wanes with time. This loss of transgene expression was also confirmed with the vaccine against endogenous TRP-1, and the loss of its expression in injected muscles coincided with the infiltration of T cells. Altogether, these results provide evidence that CTLs generated by our optimized vectors are fully functional.

Among our constructs, optTRP-1 provided the highest levels of CTLs, and its effect as a single vaccine was comparable to the effect of the multivalent vaccine. Hence, in our experimental settings, where all mice had the same immunological background, the benefits of the multivalent vaccine were not obvious. However, for application in a broad population where immunodominant epitopes differ among individuals, multivalent vaccines might have much greater therapeutic potential than a single TAA vaccine. The successful usage of the multivalent vaccine was recently published by Sahin et al*.*^40^ In a phase I clinical trial with a multivalent mRNA-based vaccine against melanoma, patients developed a strong response to different TAAs, and 75% of patients developed an immune response against at least one TAA, but most of them had a polyepitopic CD8^+^ T response.

In a therapeutic model, the optTRP-1 vaccine induced a significant delay in tumor development. The absence of a complete response could be partially explained by a lack of sufficient time for the development of vaccine-mediated immunity. B16F10 melanoma is an aggressive tumor, and a syngeneic animal model has a narrow time window for therapeutic intervention; at the same time, vaccination requires at least 2 weeks to develop an Ag-specific immune response. In addition, B16F10 is considered as poorly immunogenic or a “cold” tumor,^41^ which means that single treatment with vaccination might be not sufficient to achieve its complete eradication and would require the rational combination of several approaches/treatments.^19^^,^^42^

We performed an initial analysis of changes in intramural immune cells induced by vaccination. As expected, we observed the accumulation of TRP-1-specific CD8^+^ T cells within 2 weeks after vaccination. We also observed significant recruitment of NK cells into the tumor after vaccination. Notably, NK cells can kill tumor cells directly as well as by activating DCs through IFNγ production.^43^^,^^44^ While the vaccine should not necessarily directly affect macrophages or the MDSC population, we also found that after vaccination, intratumoral macrophages express less CD206 and more MHC class II, which indicates changes in their polarization. In summary, the observed multiple changes indicated that the administration of our vaccine induces remodeling of the intratumoral immune cell population, which can further help to design combinatorial treatments.

Our study showed that the AAV6-based vaccine successfully creates an immune response against low immunogenic self-Ags and provides a significant level of protection against tumor development. This is an important first step in successful vaccine development; however, further optimization might be necessary to increase durability and capability for boosting. The AAV vaccine could also be combined with checkpoint inhibitors or with other therapies that mitigate the immunosuppressive nature of the TME. We previously showed that the AAV-OOVA vaccine potentially benefits from combination with anti-PD-1 antibodies. Moreover, the development of vaccines based on several unrelated AAV serotypes further creates the opportunity to combine them in a prime-boost scheme of treatment, which should significantly increase vaccine potency.^45^^,^^46^

AAV vectors are a versatile platform with proven flexibility,^47^^,^^48^^,^^49^^,^^50^ and our studies demonstrated the possibility for the development of AAV-based vaccines with desired characteristics by using precise modifications and rational design.

## Materials and methods

### Animals

In all experiments, 6- to 10-week-old C57BL/6 mice were used (Jackson Laboratory, Bar Harbor, ME, USA). All manipulations with the animals were performed according to the principles of the National Research Council’s Guide for the Care and Use of Laboratory Animals and with approval by the University of Minnesota Institutional Animal Care and Use Committee (IACUC).

### Vectors

Well-described melanoma-associated Ags, which represent nonmutated melanocyte differentiation self-proteins tyrosinase, gp100, TRP-1, and TRP-2, were used as targets.^51^ The truncated sequences of these four genes, which include the immunodominant peptides,^35^^,^^52^^,^^53^ were coupled with MHC class I trafficking signals to improve the presentation of MHC class I and class II epitopes in DCs^22^ as previously described.^19^ Human sequences were used for gp-100 and tyrosinase genes, while murine sequences were used for TRP-1 and TRP-2. Additionally, the TRP1 sequence was optimized by introducing the A463M mutation to increase immunogenicity.^35^ All Ags were cloned in a self-complementary (sc) expression cassette^54^ driven by the CMV promoter and packaged in AAV6 containing a single mutation in the VP3 capsid protein at amino acid position 663 to substitute serine (S) with valine (V).^20^ The same vectors expressed firefly luciferases (AAV6-S663V-fluc) were used as a mock. Vectors were packaged in HEK293 cells and purified by iodixanol gradient followed by ion-exchange column purification as described.^19^^,^^20^ The summary of vectors used in the study is presented in Table 1.

### Cells and tissues

The B16F10 melanoma cell line (ATCC) was maintained in DMEM supplemented with 10% fetal bovine serum (Gibco), 100 U/mL penicillin, and 100 μg/mL streptomycin.

Spleens were homogenized by passing through a cell strainer, and erythrocytes were removed by applying RBC lysis buffer. Spleen single-cell suspensions were resuspended in RPMI (ATCC modification, Gibco) supplemented with 10% heat-inactivated FBS (Gibco), 100 U/mL penicillin, and 100 μg/mL streptomycin.

Harvested tumors were cut into small pieces and incubated in RPMI supplemented with 100 μg/mL collagenase D and 10 μg/mL DNase I (Roche) for 30 min at 37°C with frequent mixing. At the end of incubation, tissues were passed through a 100 μm cell strainer to obtain single-cell suspensions, and then cells were treated with RBC lysis buffer. Erythrocyte-free tumor cell suspensions were used for flow cytometry analysis.

EDTA-treated cardiac blood was centrifuged at 1,400 × *g* for 20 min to separate cells from plasma. The top layer was collected and saved as plasma, and the bottom layer was mixed with RBC lysis buffer and centrifuged at 500 × *g* for 5 min after 5 min of incubation. The RBC lysis step was repeated if the cell pellets still contained red cells. Erythrocyte-free white blood cells were used for flow cytometry analysis.

### Tumor models

The prophylactic tumor challenge model was as follows: mice were vaccinated by a single intramuscular injection with 1 × 10^10^ vg per mouse of one of each individual vaccine, opt-Tyr, opt-gp100, opt-TRP1, or opt-TRP2, or with 4× opt-Vac of total 4 × 10^10^ vg per mouse, which was a combination of four individual vaccines mixed at a ratio of 1:1:1:1 in one shot. At 2 weeks post-vaccination, mice were injected with 2 × 10^5^ B16F10 cells via the tail vein. Eighteen days after tumor challenge, the mice were euthanized, and the lungs were briefly rinsed with tap water to remove blood and were bleached overnight in Fekete’s solution as described elsewhere.^19^^,^^51^ The next day, visible B16F10 black tumor nodules were counted.

The treatment model was as follows: melanoma tumors were developed by transplanting 5 × 10^5^ B16F10 cells subcutaneously into the flank on day 0. Mice were vaccinated by a single intramuscular injection of 1 × 10^10^ vg per mouse of opt-TRP1 by two different protocols: in the first protocol, mice were vaccinated 7 days before B16F10 cell transplantation, and in the second protocol, mice were vaccinated . days after tumor transplantation. A digital caliper was used to measure the tumor size every other day, and the measurements of the perpendicular diameters were recorded. Mice were euthanized when the tumor diameter reached approximately 20 mm in either direction or when severe ulcerations developed.

### Flow cytometry and antibodies

Fluorophore-conjugated antibodies to mouse were as follows: CD45 (30-F11), CD3e (145-2C11), CD4 (RM4-5), CD11c (N418), CD11b (M1/70), Gr-1 (RB6-8C5), CD44 (IM7), MHC class II (I-A/I-E) (M5/114.15.2), F4/80 (BM8), NK1.1 (PK136), and CD19 (6D5) (all from Biolegend); CD62L (MEL-14) and PD-1 (J43) (Biosciences); CD8 (KT15) (Thermo Fisher Scientific); and custom TRP1-specific H-2K^b^ MHC class I dextramers (TAPDNLGYA, Immudex). Before staining with cell surface markers, FcRs were blocked with CD16/CD32 (2.4G2, BD Biosciences) antibodies. Dead cells were excluded by positive staining with 7-AAD (Biolegend), and all data were gated on live and single cells (FSC-A vs. FSC-H). Data were acquired using BD FACS LSR Fortessa or BD FACS Aria II, and raw data were analyzed by FlowJo software (v.10.0.3).

### ELISPOT

The presence of Ag-specific T cells in the spleens of immunized animals was measured by IFNγ production in response to restimulation with corresponding immunodominant peptides by standard ELISPOT assay (Cellular Technology, Shaker Heights, OH, USA) according to the manufacturer’s protocol. Splenocytes were used in the assay either directly after harvesting or after they were stimulated with immune peptides and cultured for 7 days in the presence of 10 ng/mL interleukin-21 (IL-21) and 25 ng/mL IL-15. For all experiments, splenocytes were seeded at a concentration of 2 × 10^5^ cells/well and stimulated with a single peptide or a pool of peptides of the corresponding Ag at a final concentration of 1 μg/mL. The peptides used in the study were the H-2Db immunodominant peptide mgp100_25-33_ EGSRNQDWL (Anaspec), H-2Db-restricted epitope mTRP1_455-463_ TAPDNLGYA (GenScript), and H-2Kb-restricted epitope mTRP2_180-188_ SVYDFFVWL (GenScript). For tyrosinase, the pool of overlapping peptides covering the full sequence of human tyrosinase was used (PepMix, JPT Peptide Technologies). Plates were cultured for 24 h and developed according to the manufacturer’s instructions. The IFNγ-secreting spots were counted with an ELISPOT reader. The results are presented as the number of spots per 1 × 10^6^ cells.

### Complement-dependent cytotoxicity (CDC) assay

Antibody-induced cytotoxicity was measured by using Cytotox green reagent (Sartorius), which enters cells with compromised membranes, and its fluorescence increases by several orders of magnitude after binding with intracellular nucleic acids. B16F10 cells were seeded in 96-well plates at a density of 10,000 cells per well in DMEM supplemented with 10% FBS. The next day, the cells were washed with PBS and loaded with 100 μL Cytotox green diluted 3,000 in serum-free DMEM. The samples of tested mouse plasma were diluted with DMEM (35 μL plasma in 300 μL DMEM) and added to assigned wells at a volume of 50 μL per well. Cells were incubated for 30 min to allow antibodies to opsonize cells. At the end of incubation, 50 μL baby rabbit complement (CEDARLANE Laboratories) diluted 4-fold with DMEM was added to each well. Cells were incubated for an additional 6 h, and CDC was estimated by the increase in Cytotox green fluorescence. The fluorescence was recorded either by a Nikon microscope (for experiments with optVac) or by an IncuCyte Live Analysis System (Sartorius) (for experiments with optTRP-1).

### Luciferase activity in injected muscles

Due to the limits in the size of the inserted gene for double-stranded AAV vectors, we chose gLuc with a size of 20 kDa over Firefly (62 kDa) or Renilla (36 kDa) luciferases as the reporter gene for *in vivo* studies. To prevent secretion, gLuc was cloned without a signal peptide in AVV6-S663V (ΔSP-gLuc) and with or without fusion with Sec and MITD (opt-ΔSP-gLuc). The activity of both constructs was first analyzed *in vitro* in HEK 293 cell lysates and in conditioned media for 48 h after infection at an MOI of 1,000 vg/cell. To compare the activity in cells and in media, 1/10 media or 1/10 cell lysate were mixed with Gaussia Flash Substrate (Thermo Fisher Scientific), and luciferase-induced chemiluminescence was measured on a BioTek spectrophotometer. To measure the gLuc activity in infected muscles, mice were injected in the gastrocnemius muscle with 5 × 10^9^ vg ΔSP-gLuc or opt-ΔSP-gLuc, and at 11 or 17 days post-injection, mice were sacrificed, and small pieces of injected muscles with a size of 3 × 3 mm were flash frozen in liquid nitrogen and stored at −80°C until use. On the day of the assay, muscle samples were homogenized in 100 μL lysis buffer, clarified by centrifugation, and 10 μL sample lysates were mixed with substrate (gLuc flash assay kit, Thermo Fisher Scientific). The chemiluminescence was measured on a BioTek spectrophotometer.

### Immunohistochemistry

Mouse muscles were dissected 1, 2, and 3 weeks after vaccination at the site of injection. Samples were embedded in OCT compound immediately after collection and frozen in liquid-nitrogen-cold butanol. Immunohistochemistry (IHC) was performed, and muscles were cut into 5 μm sections. The slides were fixed in precooled acetone (−20°C) and incubated with 3% H_2_O_2_ to block endogenous peroxidases. Sections were blocked in 10% goat serum before staining with primary antibodies against CD3 (Novus Biological, diluted 1:200) and TRP1 (Novus Biological, diluted 1:200) at 4°C overnight. Slides were incubated with biotinylated secondary antibodies for 1 h at room temperature for detection. The signal was developed by using the ABC Peroxidase Staining Kit (Thermo Fisher Scientific) following the manufacturer’s recommendations. After developing with 3,3′-diaminobenzidine (BioLegend) staining, the muscle slides were counterstained with hematoxylin and photographed using a Zeiss Axiolab 5 Histology Microscope at 20× magnification.

### Statistical analysis

All data were analyzed and plotted using GraphPad Prism software. Data are shown as the mean ± SEM. For all statistical analyses, an unpaired t test was used. Data were considered significant when p values were <0.05.

### DATA AVAILABILITY

Data and materials described in this article will be available upon request to corresponding author.
